# An Easily Fabricated Electrochemical Sensor Based on a Graphene-Modified Glassy Carbon Electrode for Determination of Octopamine and Tyramine

**DOI:** 10.3390/s16040535

**Published:** 2016-04-13

**Authors:** Yang Zhang, Meiqin Zhang, Qianhui Wei, Yongjie Gao, Lijuan Guo, Khalid A. Al-Ghanim, Shahid Mahboob, Xueji Zhang

**Affiliations:** 1 Research Center for Bioengineering and Sensing Technology, University of Science and Technology, Beijing 100083, China; zhangybslf@126.com (Y.Z.); wqhwanwan@126.com (Q.W.); gaoyongjieustb@126.com (Y.G.); 13120328993@163.com (L.G.); 2 Department of Zoology, College of Science, P. O. Box 2455, King Saud University, Riyadh 11451, Saudi Arabia; Kghanim@ksu.edu.sa (K.A.A.-G.); shahidmahboob60@hotmail.com (S.M.); 3 Department of Zoology, Government College University, Fsisalabad 38000, Pakistan

**Keywords:** octopamine, tyramine, graphene, electrochemical sensor

## Abstract

A simple electrochemical sensor has been developed for highly sensitive detection of octopamine and tyramine by electrodepositing reduced graphene oxide (ERGO) nanosheets onto the surface of a glassy carbon electrode (GCE). The electrocatalytic oxidation of octopamine and tyramine is individually investigated at the surface of the ERGO modified glassy carbon electrode (ERGO/GCE) by using cyclic voltammetry (CV) and differential pulse voltammetry (DPV). Several essential factors including the deposition cycle of reduced graphene oxide nanosheets and the pH of the running buffer were investigated in order to determine the optimum conditions. Furthermore, the sensor was applied to the quantification of octopamine and tyramine by DPV in the concentration ranges from 0.5 to 40 μM and 0.1 to 25 μM, respectively. In addition, the limits of detection of octopamine and tyramine were calculated to be 0.1 μM and 0.03 μM (S/N = 3), respectively. The sensor showed good reproducibility, selectivity and stability. Finally, the sensor successfully detected octopamine and tyramine in commercially available beer with satisfactory recovery ranges which were 98.5%–104.7% and 102.2%–103.1%, respectively. These results indicate the ERGO/GCE based sensor is suitable for the detection of octopamine and tyramine.

## 1. Introduction

**Figure 1:**
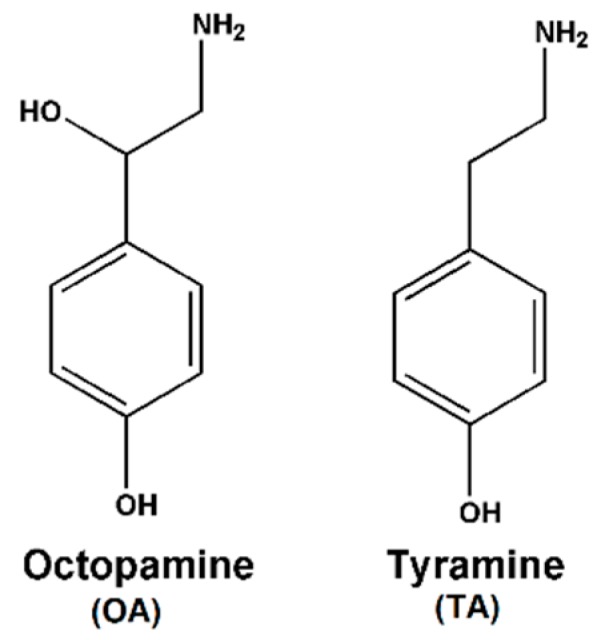
Chemical structures of OA and TA.

Octopamine (OA) and tyramine (TA) (Figure 1), are nitrogenous low molecular weight organic compounds, which are classified as biogenic amines (BAs). BAs are produced through the decarboxylation of amino acids caused by microorganisms under unhygienic conditions in some foods and beverages. High amounts of BAs are not only chemical markers for the hygienic conditions of foods and beverages but also can give rise to detrimental carcinogenic compounds [1,2,3]. However, the Food and Drug Administration (FDA) and European Union (EU) have only established legal limits for histamine in fish and fish products so far. These official organizations have not recognized the toxicity of other BAs. Except for histamine, other BAs such as OA and TA could cause considerable adverse health influences on human. On the one hand, OA and TA may enhance the toxicity of histamine by blocking the enzymatic systems which can catabolize histamine [3]. On the other hand, the ingestion of excessive amount of TA can result in nausea, hot flushes, cold sweats, palpitations, headaches, rash, high or low blood pressure and death in severe cases [4]. Till now, OA and TA have been discovered in many foods and beverages, including beer, soy sauces, fish, aged meat, sausage, wine and cheese [5,6,7,8,9]. Therefore, it is significant to develop a convenient and effective detection method for OA and TA concentrations in these foods and beverages in view of their effect on human health and food quality.

Until recently, several analytical techniques such as high-performance liquid chromatography (HPLC) and capillary electrophoresis (CE) coupled with various detectors, such as ultraviolet (UV)-vis spectroscopy, fluorospectrometry (FL) and mass spectrometry (MS) have been applied for the determination of OA and TA [10,11,12,13,14,15,16,17,18,19,20,21]. These approaches are very effective for detecting multiple analytes in complex samples owing to the excellent separating performance of HPLC or CE. However, the tedious optimization of mobile phase (pH and constitution of eluents) and time consuming and complicated elution processes limit the application of HPLC-based measurements. Furthermore, the utilization of CE coupling techniques is hindered by its poor reproducibility [22]. One the other hand, in order to improve the sensitivities, extra derivatization treatments are usually needed in UV or FL detection. Moreover, MS instrumentation is unavailable in most laboratories because the high cost of the equipment. Recently, electrochemical detection of OA and TA has attracted increasing attention ascribed to its advantages, including high sensitivity, short analysis time, simple performance, low cost and the capability of on-line and real-time analysis.

Several electrochemical biosensors for the detection of TA have been reported. A monoamine oxidase A (MAO, EC 1.4.3.4)-modified graphite electrode was used for the detection of TA [23]. In addition, a biosensor fabricated by immobilizing tyrosinase on a phosphate-doped polypyrrole film modified platinum electrode was demonstrated for TA analysis [24]. In this sensor, the polypyrrole could act both as transducer and immobilization matrix for the enzyme. In addition, several biosensors based on carbon nanotubes have been developed for TA detection [25,26,27,28]. Among them, a molecularly imprinted electrochemical sensor was constructed from a multi-walled carbon nanotube-gold nanoparticle/chitosan composites modified glassy carbon electrode (GCE) which exhibited a low limit of detection (LOD) for TA [28]. Furthermore, the cylindrical carbon fiber microelectrode (CFME) coupled with fast-scan cyclic voltammetry (FSCV) was adopted for the detection of OA and TA [29]. Recently, solid carbon nanopipette electrodes (CNPEs) with smaller diameter tips (∼250 nm) than the CFME was reported [30]. CNPEs were used to detect the electroactive neurotransmitters dopamine, serotonin and OA. All the carbon based sensors mentioned above indicate the good performance of carbonaceous materials for the electrochemical detection of OA and TA. However, to the best of our knowledge, an electrochemical sensor based on the graphene for the determination of OA and TA has not been reported so far.

The electrochemical catalysis of carbonaceous materials was studied by the research groups of McCreery and Kariuki. They demonstrated that the high electron transfer properties of carbonaceous materials could be attributed to their structural defects on the edge planes and surface oxides by investigating the electrochemical responses of various benchmark redox systems on the modified glassy carbon electrode or highly oriented pyrolytic graphite (HOPG) [31,32,33,34]. Compared to HOPG, graphene possesses better electrochemical catalysis properties due to its higher density of edge-plane-like defect sites [35]. Graphene is a closely packed honeycomb two-dimensional lattice consisting of a single layer of carbon atoms [36] that has been making a huge impact in many fields of science and technology due to its remarkable physicochemical properties such as high specific surface area [37], extraordinary electronic properties and electron transport capabilities [38,39]. For example, graphene-based electrochemical sensors have been fabricated and applied to different applications such as methanol oxidation [40], dopamine oxidation [41], single DNA sensing [42] and hydrogen evolution reactions [43]. However, most preparations of graphene modified electrodes in previous works were performed by the drop-coating method which is unable to control the thickness of films. In order to solve this problem, Chen *et al.* developed a facile and direct preparation of graphene modified glassy carbon electrodes, which was able to electrochemically reduce oxidized graphene and deposit graphene film on the electrode simultaneously by the cyclic voltammetry (CV) method [44]. In their work, the thickness of the graphene film could be easily controlled by modifying the CV deposition cycles.

Herein, based on the work of Chen *et al.* [44], we fabricated a simple electrochemical sensor for the detection of OA and TA based on electrochemically reduced graphene oxide nanosheets (ERGO)-modified GCE. After the optimization of several key factors, this sensor showed a wide linear range, low LOD, satisfactory reproducibility, good selectivity and stability for the determination of OA and TA. Furthermore, it was successfully applied to the analysis of OA and TA in beer.

## 2. Experimental

### 2.1. Chemicals and Reagents

Octopamine hydrochloride (>95% purity) was obtained from Sigma (Shanghai, China). Tyramine hydrochloride with 98% purity was purchased from Alfa Aesar (Shanghai, China). Graphite oxide with 99% purity was purchased from Nanjing XFNANO Materials Tech Co., Ltd. (Nanjing, China). Phosphate buffers (PB, 0.1 M) at different pH values (5.0, 5.5, 6.0, 6.5, 7.0, 7.5, 8.0) were prepared by mixing 0.1 M KH_2_PO_4_ with KOH. All other chemicals, bought from Sinopharm Chemical Reagent Beijing Co., Ltd. (Beijing, China), were of analytical grade purity and used as received. Triple distilled water was used throughout this work. Tsingtao beer (LOT: 20131220 01611:35) purchased from a local supermarket was used as the real sample without any pretreatment.

### 2.2. Instruments

All electrochemical measurements were carried out using a CHI440 electrochemical workstation (Chenhua Instrument Company, Shanghai, China) equipped with a conventional electrochemical cell containing a three-electrode system. A bare GCE (diameter: 3 mm) or ERGO/GCE, Ag/AgCl (3 M KCl) and a platinum wire were used as working, reference and auxiliary electrode, respectively. The scanning electron microscopic (SEM) characterization was performed with a NOVA NanoSEM 450 microscope (FEI, Eindhoven, The Netherlands). All experiments were carried out at room temperature (23 ± 2 °C).

### 2.3. Preparation of ERGO/GCE

The preparation of the ERGO/GCE employed the experimental procedures reported by Chen *et al.* [38]. The graphene oxide (GO) colloidal dispersion solution was prepared by dissolving 40 mg graphite oxide powder in 20 mL 0.07 M Na_2_HPO_4_ solution (pH 9.2) and then sonicating for 2 h. Prior to the electrodeposition experiments, the GCE was polished using 0.3 and 0.05 µm diameter alumina slurry and subsequently sonicated in acetone, ethanol and water for 1 min. After immersing the clean bare GCE into the GO solution vertically, the electrodeposition of ERGO on the surface of GCE was performed by cyclic voltammetric (CV) method with different scanning cycles (3, 6, 9, 12, 15, 18 and 21 cycles). The potential range of successive CV negative scanning was from 0.65 to −1.5 V with a scan rate of 50 mV·s^−1^. After the modification, the ERGO/GCE was then thoroughly rinsed with water and dried in air.

### 2.4. Analytical Procedure

The determination of OA and TA were performed by CV and differential pulse voltammogram (DPV) method. The potential scanning range for CV experiments was from 0.4 V to 0.8 V with the scan rate of 0.1 V·s^−1^. The DPV parameters for OA analysis were chosen as follows: amplitude of 80 mV, pulse width of 40 ms and pulse repetition time of 0.1 s. For TA detection, the amplitude, pulse width and pulse repetition time were 100 mV, 50 ms and 0.3 s, respectively. If not stated otherwise, 0.1 M·PB (pH 7.0) was used as the supporting electrolyte solution.

## 3. Results and Discussion

### 3.1. Electrodeposition of Graphene Nanosheets

**Figure 2:**
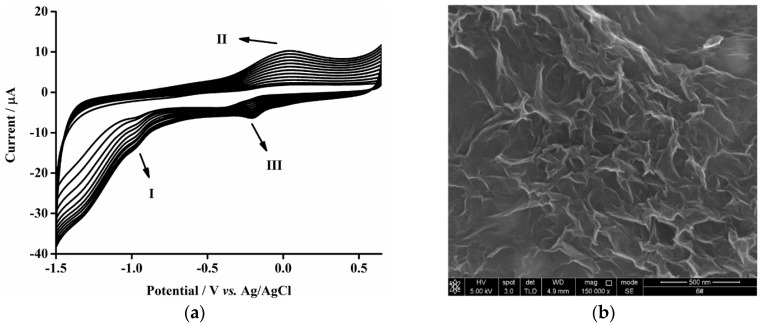
(**a**) CV of bare GCE in 0.07 M Na_2_HPO_4_ aqueous solution (pH 9.2) containing 2.0 mg·mL^−1^ GO. The scan cycle: 12 cycles; potential range: −1.5 to 0.65 V; scan rate: 50 mV·s^−1^. (**b**) The SEM image of ERGO/GCE.

The characteristic CV of electrodeposition of ERGO on the GCE is shown in Figure 2a. One anodic peak (II) and two cathodic peaks (I and III) can be observed during the scanning. The gradual increase of the peak currents with successive CV scanning reveals the successful electrodeposition of graphene nanosheets on the electrode. Peak I is ascribed to the irreversible electrochemical reduction of GO and the other pair of peaks (II and III) are attributed to the redox of the electrochemically active oxygen-containing functional groups on the planes of the depositing graphene nanosheets [44]. Moreover, the surface morphology of ERGO film on the GCE is further characterized by SEM shown in Figure 2b, which presents the wrinkly lamellar structure of graphene nanosheets. In addition, the ERGO film is insoluble and reasonably stable in aqueous solution [44].

### 3.2. Electrochemical Detection of OA and TA with ERGO/GCE

**Figure 3:**
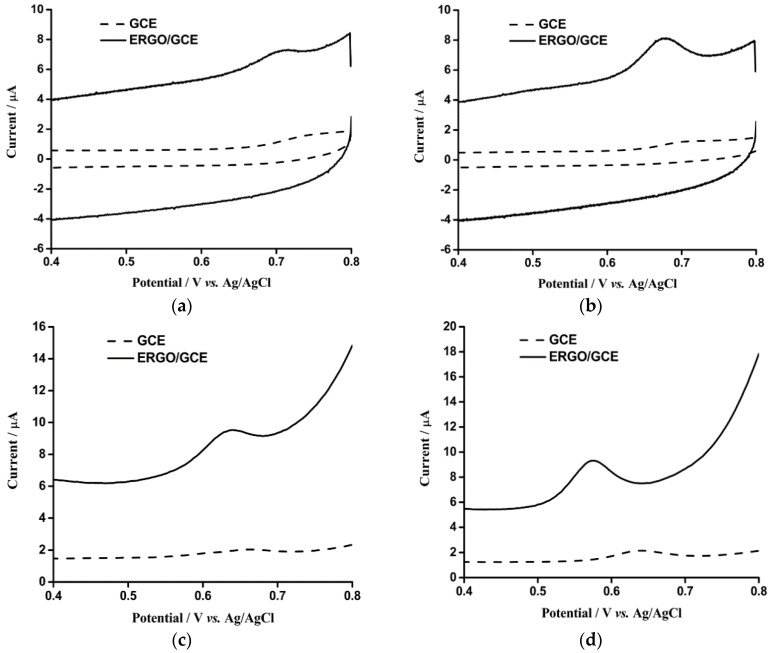
(**a**,**b**): CV of GCE (dash line) and ERGO/GCE (solid line) in 0.1 M phosphate buffer solution (pH 7.0) containing 5 μM (OA) (**a**) and 5 μM (TA) (**b**). The potential range: 0.4 to 0.8 V; scan rate: 100 mV·s^−1^. (**c**,**d**): DPV of GCE (dash line) and ERGO/GCE (solid line) in 0.1 M phosphate buffer solution (pH 7.0) containing 5 μM (OA) (**c**) and 5 μM (TA) (**d**). The potential range: 0.4 to 0.8 V; amplitude: 80 mV (OA) and 100 mV (TA); pulse width: 40 ms (OA) and 50 ms (TA); pulse repetition time: 0.1 s (OA) and 0.3 s (TA); the ERGO/GCE was modified by scanning 12 CV cycles.

The electrochemical behaviors of OA (5 μM) and TA (5 μM) at bare GCE and ERGO/GCE were firstly investigated by performing CV from 0.4 to 0.8 V in a supporting electrolyte solution of 0.1 M PB (pH 7.0). The obtained CV curves of 5 μM OA and 5 μM TA on these two different electrodes are shown in Figure 3a,b, respectively. The electrochemical reaction of OA and TA were irreversible on these two kinds of electrodes because only the oxidation peak could be observed. At the bare GCE (dashed curves), OA and TA had oxidation peak potentials at 730 and 700 mV, respectively. Moreover, the anodic peak potential of OA and TA at the ERGO/GCE (solid curves) shifted towards more negative values than that obtained at the bare GCE, which were at 700 mV for OA and 680 mV for TA. On the other hand, the oxidation peak currents of OA and TA at the ERGO/GCE were dramatically enhanced in comparison with the bare GCE. It was calculated that the anodic peak currents of OA and TA at the ERGO/GCE were 13.6 and 19.4 times than those at the bare GCE. Besides, it is worthwhile to note that the background current of the ERGO/GCE also increased significantly, which might be ascribed to the larger surface area of the ERGO deposits on the GCE [44]. Furthermore, owing to the better sensitivity of DPV technique than the CV [44], DPV detections of OA (Figure 3c) and TA (Figure 3d) were performed under the same experimental conditions. The similar negative shifts of the oxidation peak potentials and considerable enhancement of the anodic peak currents for both OA and TA were observed as well. For OA, the oxidation peak potential shifted negatively from 660 to 630 mV and the peak current increased 13.7 times. In addition, the negative shift of oxidation peak potential for TA was from 640 to 570 mV and the corresponding peak current raised about 16 times. The easier oxidation and larger oxidation peak currents of OA and TA sensing at the ERGO/GCE than the bare GCE owing to the large surface area, good electron conductivity and the residual oxygen-containing active groups of the graphene nanosheets [45,46].

### 3.3. The Effect of CV Scanning Rate and pH on the Electrochemical Oxidations of OA and TA on ERGO/GCE

**Figure 4:**
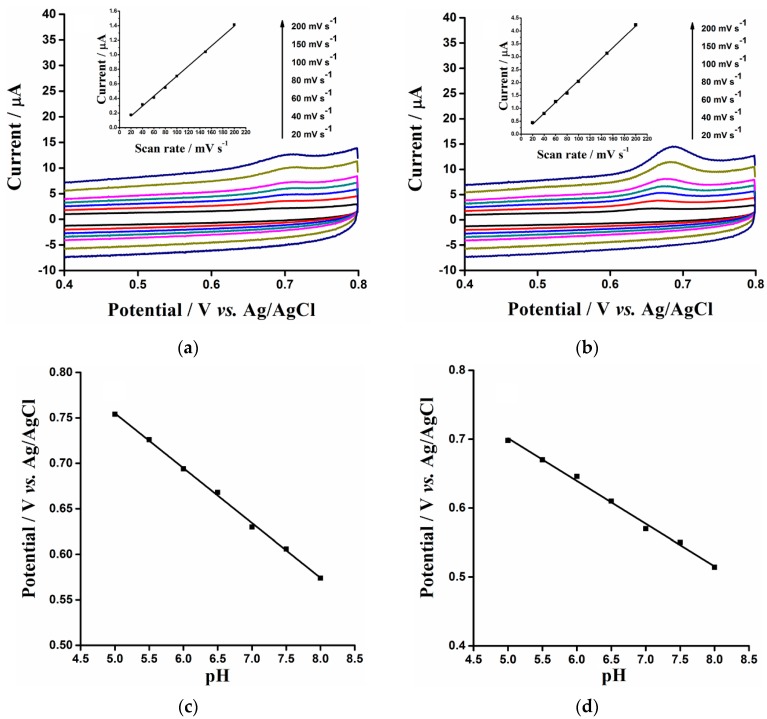
(**a**,**b**): CV of ERGO/GCE in 0.1 M phosphate buffer solution (pH 7.0) containing 5 μM (OA) (**a**) and 5 μM (TA) (**b**) with different scan rates (20, 40, 60, 80, 100, 150 and 200 mV·s^−1^). The potential range: 0.4 to 0.8 V. Insets: the corresponding linear relationships between peak current and scan rate. (**c**,**d**): The linear relationship between pH of phosphate buffer solution (5.0, 5.5, 6.0, 6.5, 7.0, 7.5 and 8.0) and DPV anodic peak potential (*E_pa_*) of 5 μM (OA) (**c**) and 5 μM (TA) (**d**).

The relationship between the peak current and scan rate of potential in CV could provide information about the mechanism of the analytes’ electrochemistry. The CVs of OA (Figure 4a) and TA (Figure 4b) at the ERGO/GCE in 0.1 M PB (pH 7.0) were recorded at different scan rates (from 20 to 200 mV·s^−1^) for investigating the kinetics of their oxidation reactions. With increasing scan rate (*v*), the anodic peak currents (*I_pa_*) of both OA and TA increased accordingly and a good linear plot of *I_pa_ vs. v* was acquired with a *R*^2^ value of 0.997 and 0.999 for OA and TA, indicating the kinetics are an adsorption-controlled process of electro-oxidation of OA and TA on the surface of the ERGO/GCE.

In order to verify the participation of the proton in the electrochemical oxidation of OA and TA, the influence of the pH of the supporting electrolyte was investigated. The anodic peak potentials (*E_pa_*) of 5 μM (OA) and 5 μM (TA) in 0.1 M PB with a pH range from 5.0 to 8.0 were shown in Figure 4c,d, respectively. As expected, the *E_pa_* of OA and TA shifted negatively with the increase of pH value, indicating that protons took part in the electrochemical reaction. The relationship of *E_pa_* and pH of PB solution for OA and TA is linear. The corresponding expression of OA is:

(1) *E_pa_* (*v*) = 1.056 − 0.0602 pH (*R*^2^ = 0.998)

and the expression for TA is:

(2) *E_pa_* (*v*) = 1.0112 − 0.062 pH (*R*^2^ = 0.994)

**Figure 5:**
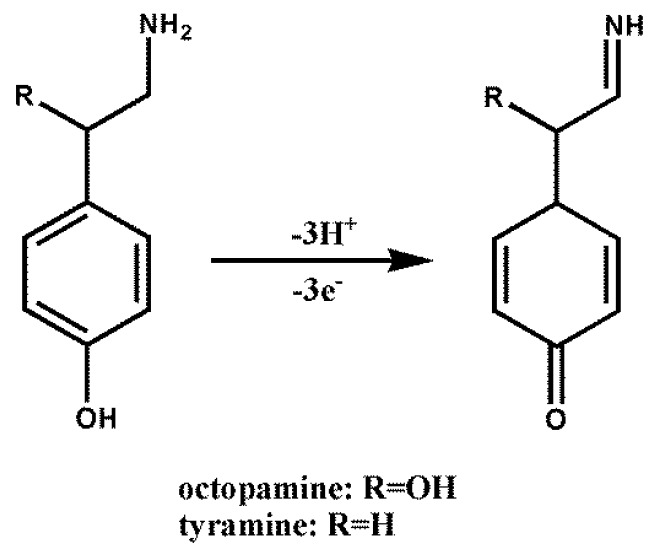
Possible reaction for electrochemical oxidation of OA and TA at the surface of ERGO/GCE.

The value of the slopes for OA and TA is close to an expected Nernstian slope. On the other hand, according to the following formula [47]:

(3) $$\frac{dE_{pa}}{dpH}=\frac{-2.303\;\text{mRT}}{\text{nF}}$$

where m and n are the participating number of protons and electron transfer number in the reaction respectively. The ratio of m/n at ERGO/GCE was calculated to be approximately 1, suggesting that the equal numbers of protons and electrons were transferred in both electrochemical oxidation reactions of OA and TA. The results were consistent with the reported electrochemical mechanism that three protons and three electrons are involved in the electrochemical oxidation of OA and TA [26,48,49]. Figure 5 illustrates a possible process for electrochemical oxidation of OA and TA at the surface of ERGO/GCE.

### 3.4. Optimization of Key Parameters for Electrochemical Oxidations of OA and TA

**Figure 6:**
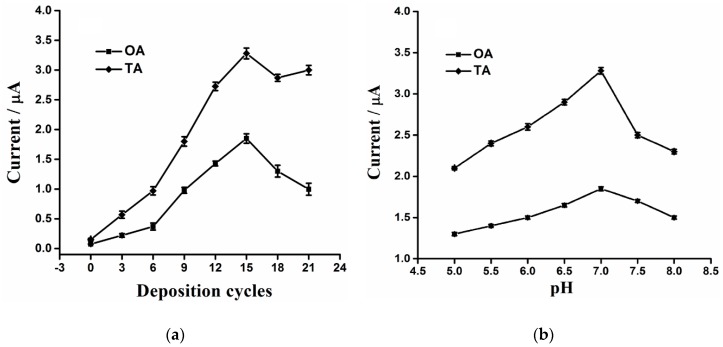
(**a**) The influence of graphene depositing cycles (0, 3, 6, 9, 12, 15, 18 and 21 cycles) on the DPV anodic peak currents of 5 μM (OA) and 5 μM (TA); (**b**) The influence of electrolyte solution pH (5.0, 5.5, 6.0, 6.5, 7.0, 7.5 and 8.0) on the DPV anodic peak currents of 5 μM (OA) and 5 μM (TA).

In order to maximize the anodic peak currents of OA and TA at the ERGO/GCE, we optimized two vital parameters including the depositing cycle of ERGO and the pH of PB. The relationships of the ERGO depositing cycles *vs.* the anodic peak currents of 5 μM OA and 5 μM TA were shown in Figure 6a.

When the number of ERGO deposition cycles was gradually increased from 0 to 15 cycles, the anodic peak currents of both OA and TA were enhanced. The possible reason is that the increase of the ERGO leads to a larger specific surface area and more active sites for OA and TA. When the number of deposition cycles was further increased to 21 cycles, the anodic peak currents decreased greatly, which may be due to the close packed structure of excess graphene nanosheets at the ERGO/GCE that reduces specific surface area and hinders the mass transfer [50]. Hence, 15 deposition cycles was selected for fabricating the ERGO/GCE sensor. In addition, the relationship between the anodic peak currents of OA and TA and pH of PB was also investigated and the results are shown in Figure 6b. It could be observed that pH 7.0 was the optimal condition for the electrochemical oxidation of both analytes.

### 3.5. Reproducibility, Linear Range and Limit of Detection

**Figure 7:**
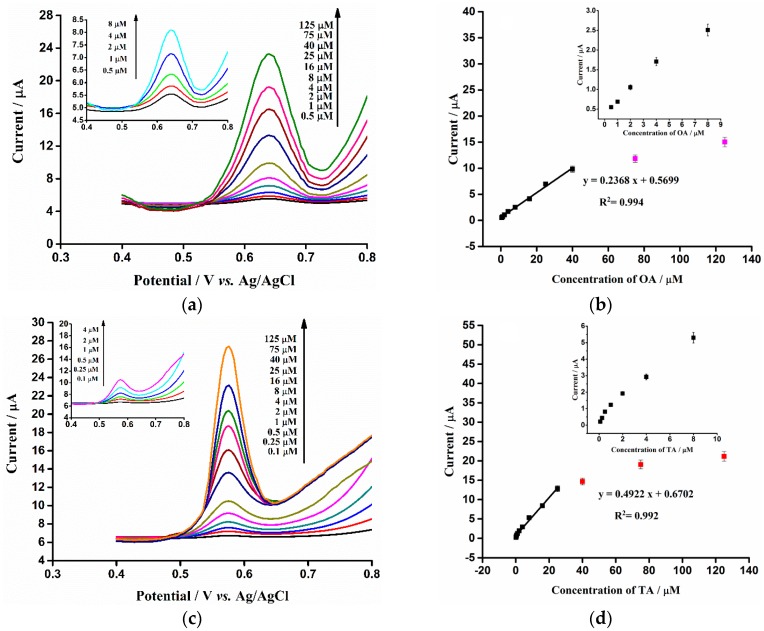
(**a**,**c**): DPV of ERGO/GCE in 0.1 M phosphate buffer solution (pH 7.0) containing different concentrations of OA (**a**) and TA (**c**). The DPV parameters of OA and TA were the same as Figure 3c,d, respectively; (**b**,**d**): The corresponding relationships of DPV anodic peak currents *vs.* the different concentrations of OA (**b**) and TA (**d**).

The reproducibility, linear range and LOD of the fabricated ERGO/GCE sensor were also studied by the DPV method under the optimum experimental conditions. The reproducibility was evaluated by the detection of 5 µM (OA) and 5 µM (TA) with ten independent ERGO/GCEs. Relative standard deviations (RSDs) of 6.7% and 6.6% were obtained for OA and TA, respectively. The results indicated that this ERGO/GCE electrochemical sensor possesses good reproducibility. Moreover, the linear concentration range of OA and TA were investigated through the DPV experiment of the ERGO/GCE in electrolytic solutions containing different concentrations of OA (Figure 7a) and TA (Figure 7c). The analytical plot of the anodic peak current of OA and TA *vs.* their concentration is shown in Figure 7b,d, respectively. The linear concentration ranges of OA and TA were 5.0 × 10^−7^ to 4.0 × 10^−5^ M and 1.0 × 10^−7^ to 2.5 × 10^−5^ M, respectively. The LODs (S/N = 3) were calculated to be 100 and 30 nM for OA and TA, respectively, which were comparable [28,29] or even better [23,24,25,26,27] than the values in Table 1 reported by other research groups.

### 3.6. Interference Studies

**Figure 8:**
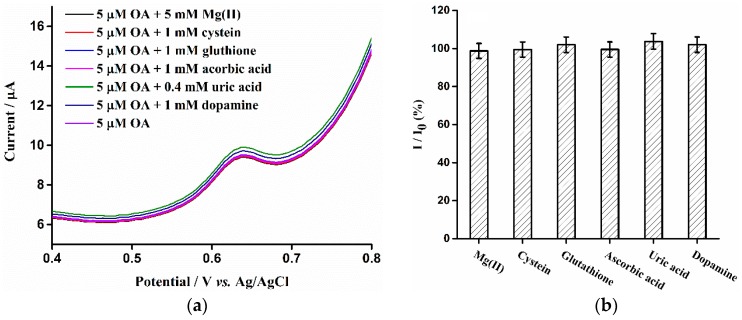

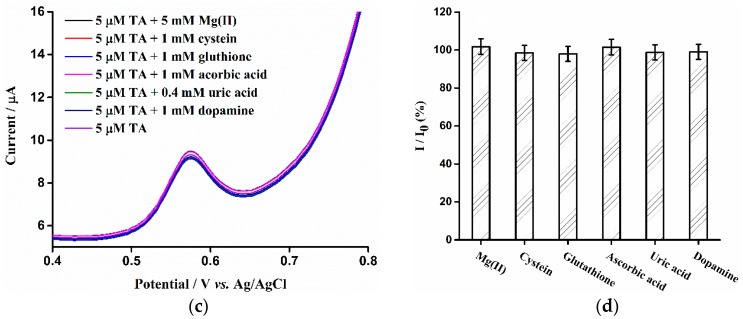
(**a**,**c**): DPV of ERGO/GCE in in 0.1 M phosphate buffer solution (pH 7.0) containing 5 µM (OA) (**a**) and 5 µM (TA) (**c**) in the presence of interferents (5 mM·Mg (II), 1 mM cysteine, ascorbic acid, dopamine and glutathione, and 0.4 mM uric acid). The DPV parameters of OA and TA are the same as Figure 3c,d, respectively; (**b**,**d**): The effect of interferents on the DPV anodic peak current of OA (**b**) and TA (**d**). I and I_0_ represent the DPV anodic peak current before and after the addition of interferent, respectively.

The interferences resulting from foreign substances on the oxidation peak current of OA and TA at the surface of ERGO/GCE sensor under the optimum conditions was also studied by DPV measurements. Referring to the works of Raoof’s group [26,27], possible interfering substances which are abundant in biological samples including Mg^2+^, cysteine, ascorbic acid (AA), dopamine and glutathione and uric acid (UA) were selected. As shown in Figure 8a,c, no significant change of the DPV anodic peak current of OA and TA in the presence of interferents was observed. Besides, the ratio (%) between the oxidation peak current of OA (Figure 8b) and TA (Figure 8d) in the presence (I) and absence (I_0_) of the interferences is in the range of 95% to 105%, which further indicates that the interferences have almost no influence on the oxidation peak current of OA and TA at the surface of the sensor. In these experiments, the concentrations of interfering substances used in the detection of 5 μM (OA) and TA were 5 mM for Mg^2+^, 1 mM for cysteine, AA, dopamine and glutathione, and 0.4 mM for UA. It proves that ERGO-GCE can be regarded as a good electrochemical sensor for recognition of OA and TA.

### 3.7. Stability

The stability of a sensor is an essential factor to be evaluated. Therefore, sensor stability experiments were performed by evaluating the oxidation peak currents of 5 μM (OA) and TA at regular intervals (1 d) for a period of 2 weeks. The results showed that 95% of the initial oxidation peak current signal was retained after successive determinations for 2 weeks, suggesting the sensor had good stability.

### 3.8. Real Sample Analysis

As described above, OA and TA have been discovered in many foods and beverages such as beers [13]. Thus, in order to illustrate the ERGO/GCE sensor application in practical analysis, a commercial beer was chosen for the determination of OA and TA with the DPV method. The standard addition method was adopted and the results are shown in Table 2. The recovery values ranged from 98.5% to 104.7% for OA and 102.2% to 103.1% for TA with the RSD (*n* = 5) less than 8%, indicating that this sensor is reliable for the quantification of the content of OA and TA in beer.

## 4. Conclusions

We have developed a sensor based on a graphene nanosheet-modified glassy carbon electrode prepared through a one-step electrochemical deposition method. The electrochemical behaviors of OA and TA at the surface of ERGO/GCE were studied by CV and DPV measurements. The anodic signals of OA and TA were enhanced due to the large surface area, high adsorption ability and more active sites. Under the optimized experimental conditions, the sensor exhibited good selectivity, sensitivity, reproducibility and stability. In addition, low LODs within a wide concentration range were obtained compared with other sensors. The sensor has also been used for the voltammetric determination of the content of OA and TA in beer. The results obtained indicated the usefulness of the proposed device for measuring OA and TA in real samples.

## Figures and Tables

**Table 1:** Comparison of the proposed ERGO/GCE with other reported electrodes for the determination of OA and TA.

Electrode	Technique	Linear Range (μM)	LOD (S/N = 3)	Refs.
Monoamine oxidase A/graphite electrode	Amperometry	TA: 10–500	2 μM	[23]
Tyrosinase/polypyrrole/Pt electrode	Amperometry	TA: 4–80	0.547 μM	[24]
Tyrosinase/Carboxyl functionalised SWCNTs	Amperometry	TA: 5–180	0.62 μM	[25]
Carboxyl functionalised MWCNT/GCE	DPV	TA: 1–17 and 17–85	TA: 0.42 μM	[26]
Quercetin/carboxyl functionalised MWCNT/GCE	DPV	TA: 0.7–75	TA: 0.647 μM	[27]
Carbon nanofiber	Fast CV	OA and TA: 0.2–5	OA: 30 nM	[29]
			TA: 18 nM	
MWCNT-AuNP/chitosan/GCE	Amperometry	TA: 0.108–10	TA: 57 nM	[28]
ERGO/GCE	DPV	OA: 0.5–40	OA: 100 Nm	This work
		TA: 0.1–25	TA: 30 nM	

**Table 2:** Determination of OA and TA in beer samples using ERGO/GCE (*n* = 5).

Analyte	Sample	Add (μM)	Detected (μM)	Recovery (%)	RSD (%)
OA	1	5	4.925	98.5	5.9
	2	30	31.41	104.7	6.1
TA	1	5	5.11	102.2	5.6
	2	10	10.31	103.1	6.4
